# Essential oil from the roots of *Paeonia lactiflora pall.* has protective effect against corticosterone-induced depression in mice via modulation of PI3K/Akt signaling pathway

**DOI:** 10.3389/fphar.2022.999712

**Published:** 2022-09-16

**Authors:** Jia-Yi Sun, Yi-Tong Liu, Sheng-Nan Jiang, Peng-Mei Guo, Xin-Yu Wu, Jia Yu

**Affiliations:** ^1^ Chengdu University of Traditional Chinese Medicine, Chengdu, China; ^2^ The Third Affiliated Hospital of Chengdu University of TCM/Chengdu Pidu District Hospital of Traditional Chinese Medicine, Chengdu, China

**Keywords:** depression, apoptosis, PI3K-Akt pathway, oxidative stress, neuroprotection, experimental techniques

## Abstract

For thousands of years, the roots of *Paeonia lactiflora* Pall (PLP) has been considered by traditional Chinese medicine as a drug that can improve mental or emotional disorders, including depression, anxiety and affective disorders. Unfortunately, the research on the mechanism of action and active ingredients of this beneficial drug is not comprehensive. This study focused on the activity of essential oil from PLP (EOP), systematically studied the antidepressant effect of EOP for the first time, and discussed the potential mechanism of its antidepressant effect. In this study, we used a mouse model of corticosterone (CORT)-induced depression, and found that EOP had a significant antidepressant effect on the symptoms of CORT-induced depression in mice, and significantly down-regulated the levels of CRH, ACTH and cortisol in the brain tissues of mice. In addition, we found that EOP treatment alleviated CORT-induced hippocampal neuron injury in mice *In vitro* experiments. It was also found that EOP could inhibit CORT-induced apoptosis and improve the proliferation ability and cell viability of PC12 cells. Further, with the help of network analysis, it was revealed that PI3K-Akt might be one of the main signaling pathways of EOP against CORT-induced hippocampal neuron apoptosis. In this study, we also found that EOP up-regulated the phosphorylation of PI3K and Akt in CORT-induced mouse hippocampal neurons and PC12 cells, and promoted the nuclear transcription of Nrf2 in CORT-induced PC12 cells. In conclusion, with the integrated approach, we demonstrated that EOP exerted anti-apoptotic effects on hippocampal neurons through PI3K/Akt/Nrf2 signaling pathway.

## 1 Introduction

Depression which characterized by increased negative and depressed mood and decreased positive anhedonia, is one of the most common presenting clinical psychiatric symptoms worldwide (McCarron et al., 2021; Xu et al., 2021). Owing to the increasing pressure from the fast-paced life and work, the prevalence of depression remains high with over than 15% of the people worldwide (Malhi and Mann, 2018; Yu et al., 2021). Depression would bring heavy burdens to individuals and their families in health, economy, work, and interpersonal relationships, *etc*. Worse still, depression is prone to suicidal tendencies, which attracts considerable attention from the society (Oh et al., 2019; Yu et al., 2021). Although nowadays, some breakthroughs have been achieved in diagnosis of depression and clarification of its underlying pathological mechanisms, the therapeutical effect of depression remains unsatisfactory (Malhi and Mann, 2018). The current used therapies of depression include psychological therapy and pharmacotherapy. However, the psychological therapy has limited effect on depression, and patients are often reluctant to administration of synthetic antidepressants due to some anticipated side effects including drowsiness, dry mouth, gastrointestinal irritation, constipation, and sexual dysfunction, *etc*. (Han et al., 2013; Malhi and Mann, 2018) Consequently, finding more effective candidate drugs for treating depression with less toxicity is still urgently needed now.

In recent years, increasing evidences have suggested that natural herbal medicines may be one of the most attractive sources for finding new drugs with lower side effects (Atanasov et al., 2021; Yao et al., 2021), and especially, previous researches have reported lots of neuroprotective agents from the extracts/monomers of the natural herbal medicines (Peng et al., 2022). *Paeonia lactiflora* Pall (PLP) is one of the most commonly used medicinal plants in China (Xu et al., 2011; Kim et al., 2021; Sun et al., 2021). The roots of *P. lactiflora* is a well-known herbal medicine called “*Shaoyao*” in China with functions of “*Nourishing blood and regulating menstruation, Astringe Yin ang stoping sweating, Soften the liver and relieving pain, Relieving Qi stagnancy in liver and Calming the liver and suppressing yang*”, and commonly used in some famous herbal medicine formulas for treating depression, such as “*Xiaoyao San*”, “*Danggui Shaoyao San*”, “*Shaoyao Gancao Tang*” (Xu et al., 2011; Feng et al., 2021), *etc*. Previous investigations have revealed that Paeoniflorin, a monoterpenoid glycoside, is one of the active components in the roots of *P. lactiflora* with anti-depression and neuroprotective effects (Wang et al., 2021; Peng et al., 2022). However, the anti-depression properties of essential oil of the roots *P. lactiflora* (EOP) have not been reported based on systemically *in vivo* and *in vitro* experiments. In our preliminary experiment for screening potential neuroprotective agents from natural herbs, we found that the EOP had obvious neuroprotective effects on H_2_O_2_ induced PC12 cells. Therefore, in our present study, we aim to systemically evaluate the anti-depression effects of EOP based on depressive animals and cells, and further explore the possible molecular mechanisms using an integrated approach based on network analysis combined with experimental verification *in vivo* and *in vitro*.

## 2 Materials and methods

### 2.1 Chemicals, reagents, and materials

The Trans-Serum^®^ EQ Fetal Bovine Serum (FBS) was purchased from TransGen Biotech (Beijing, China); Corticosterone (CORT) was purchased from Shanghai Aladdin Biochemical Technology Co., LTD; NE, ACTH, 5-HT, CORT, DA, CRH test kits were purchased from Quanzhou Ruixin Biological Technology Co., LTD. The CCK8 kit was purchased from Wuhan Brostech Bio; AO-EB kit was obtained from Beyotime (Haimen, China); JC-1 probe, DHE probe and apoptosis kit were obtained from US Everbright^®^ Inc. Primary antibodies of phosphorylation (p)- phosphatidylinositol three kinase (PI3K), protein kinase B (AKT), PI3K, p-Akt, Nrf2, β -actin were purchased from Wuhan Abclonal Biotechnology Co., LTD; Secondary antibodies for WB and IF were obtained from CST.

### 2.2 Extraction of EOP

PLP was purchased from Chengdu Lotus Pond Medicinal herbs market and identified as the root of *Paeonia lactiflora Pall.* by Professor Jia Yu of Chengdu University of Traditional Chinese Medicine. The samples of *Radix Paeoniae Alba* were stored in the herbarium of Chengdu University of Traditional Chinese Medicine, and the sample change was RPA001. The 200 g of the medicinal material was crushed, and then eight times the amount of water was added to soak for 30min. The essential oil in the *Radix Paeoniae Alba* (EOP) was extracted with the volatile oil extractor, and the yellow and white oily liquid was finally obtained. After drying with anhydrous sodium sulfate, it was sealed and stored in -20°C for reserve.

### 2.3 Animals and interventions

60 ICR male mice (6–8 weeks, 18–22 g) were purchased from the experimental animal center of Beijing Sibef Biotechnology Co., LTD. The animal studies were approved by the Animal Care and Ethics Committee of Chengdu University of Traditional Chinese Medicine (No. 2021-03-2). They were reared in an environment of 23 ± 2°C, 55% ± 5% humidity and subjected to a conventional light-dark cycle for 12 h. The mice were fed adaptively for a week before the experiment began. Throughout the experiment, the mice were given free access to food and water. All animal care and laboratory procedures are approved by the ethics committee of Chengdu University of Chinese Medicine. All mice were randomly divided into six groups (n = 6): Control group, CORT group, Fluoxetine (3 mg/kg), EOP low-dose group (1 mg/kg), EOP medium-dose group (3 mg/kg) and EOP high-dose group (3 mg/kg). Except for the control group, mice in other groups were intraperitoneally injected with CORT 20 mg/kg daily for four consecutive weeks. From the fourth week, CORT injection was followed by daily intragastric administration of Fluoxetine and EOP, and the control group was intragastric administration of the same amount of CMC-Na for four consecutive weeks.

### 2.4 Behavior testing

All the behavioral tests were performed between 20:30 and 06:30 and the observers were blinded, meaning the behavioral test participants were unaware of the treatment the mice were receiving. Tail suspension test (TST) and forced swimming test (FST) were conducted 2 days before the end of the experiment, and sucrose preference test (SPT) and open pit test (OFT) were conducted 1 day before the end of the experiment (Yu et al., 2021).

#### 2.4.1 TST

A medical tape was affixed 2 cm from the tip of the tail of the mice, and the mice were hung separately on the top of a box-shaped device (17.5 cm × 19 cm x 32 cm). The mice were adapted to the device for 2min and then suspended for 5min, and the instrument recorded the rest time of the mice. At the end of each test, 75% ethanol was used to clean the feces around the device and wipe the instrument to remove the effect of feces or odor on the behavior measurement.

#### 2.4.2 FST

The FST was carried out on mice according to the experimental plan of X et al. In simple terms, mice were placed in cylindrical containers (11 cm in diameter and 25 cm in height), with a water depth of about 20 cm and a water temperature of 25 ± 1°C. FST was performed on mice after 2 min of adaptation in water, and the swimming conditions in the following 4 min were recorded. The mice were defined as immobile when they floated on the water surface without struggling but kept breathing only with their noses exposed, and the immobile time of forced swimming was recorded.

#### 2.4.3 SPT

SPT is often used to assess anhedonia in rodents. Simply put, the mice were deprived of food and water for 12 h before the experiment. The mice were then placed in separate cages and given free access to the solution in two bottles (one containing tap water and the other containing a 1% sucrose solution (W/V)). After 12 h, change the position of the two bottles to avoid the effect of different placement of the bottles on the results. After 24 h, the liquid consumption in both bottles was weighed and calculated. Sucrose preference is calculated according to the following formula:

Sucrose preference (SP) % = sucrose consumption (ml)/[sucrose consumption (ml) + water consumption (ml)] x 100.

#### 2.4.4 OFT

The 24 h after the last administration, mice were subjected to OFT experiment to evaluate the therapeutic effect of EOP intervention on depressed mice, and autonomous exercise, exploration and anxiety were measured. The mice were placed in a 50 cm × 50 cm black square with a 50 cm high black wall, which was divided into 16 equal areas. The movement track of the mice, and the times and time of crossing the central grid were recorded within 6 min. After the experiment, each mouse should clean the device and wipe with alcohol to prevent the residual odor from affecting the results of the experiment.

### 2.5 Sample selection

At the end of the experiment, the mice were anesthetized with pentobarbital sodium and their blood was collected by orbital blood sampling. The blood was coagulated at room temperature for 30 min. The serum was separated by centrifugation and stored at -20°C. Three mice from each group were randomly selected and injected with PBS through the heart. The skull of mouse was opened, the whole brain tissue was removed, and the right brain was fixed in 4% paraformaldehyde. The fixed samples were used for subsequent HE staining, Niger staining and immunofluorescence analysis. The brain tissues of other mice were stored in -80°C for subsequent experiments.

### 2.6 Pathological observation and morphological observation of brain tissue

The fixed brain tissue was sectioned and stained. HE staining was used to observe the pathological changes of brain tissue, and Nissl staining was used to observe the number, structural integrity and cell fullness of neurons in the hippocampus of mice.

### 2.7 Neurotransmitter assay

The frozen brain tissue was taken out, the prefrontal cortex and hippocampus were separated, and tissue lysate was added to homogenate the tissue. The tissue fluid after lysis was centrifuged at 10,000 r at 4°C for 20min. Then the supernatant was collected to obtain brain tissue protein. The levels of dopamine (DA), 5-hydroxytryptophine (5-HT), norepinephrine (NE), adrenocorticotropic hormone (CORT), adrenocorticotropic hormone (ACTH), and adrenocorticotropic hormone releasing hormone (CRH) in the lysed brain tissue were determined according to the standard steps of the commercial kit instructions.

### 2.8 Cell culture

PC12 cell lines were obtained from Guangzhou Genio Biological Company. Cells were cultured in DMEM medium containing 10% FBS in a 37°C, 5% CO2 incubator. Fresh media were replaced every 2 days and subculture was carried out every 3 days. All *in vitro* experiments were performed in triplicate biological replicates.

### 2.9 CCK-8 assay

The suitable CORT dose was screened by CCK-8 assay. The cells in the logarithmic growth phase were seeded into 96-well plates. When the cells covered 80%–90% of the bottom surface of the plates, CORT of 50–1600 μM was added and incubated with the cells for 24 h. At the end of the experiment, CCK-8 reagent was added to detect the survival rate of cells. The survival rate of different concentrations of EOP (5 μg/ml-160 mg/ml) was measured at 24 h by the same method. Similarly, the survival rate of PC12 cells after EOP treatment 24 h in the presence of CORT was also tested.

### 2.10 Acridine orange (AO) - Ethidium bromide (EB) staining

At the end of the intervention, cells were stained with AO-EB dye for 5min. At the end of the staining, intracellular fluorescence changes were observed with confocal microscopy.

### 2.11 Cell mitochondrial membrane potential (MMOP) detection

The changes of intracellular MMOP were detected by JC-1 probe. When the MMOP of cells was at normal level, JC-1 would exist in cells as aggregates. At this point, JC-1 will glow red. When MMOP decreases, JC-1 exists as a monomer and emits green fluorescence. Therefore, the change of MMOP can be evaluated by observing the change of red-green fluorescence ratio in cells. At the end of treatment, JC-1 probe was added into a small dish and incubated for 30min away from light. At the end of staining, the fluorescence changes in the cells were observed by confocal microscopy.

### 2.12 Apoptosis detection

Flow cytometry was used to detect apoptosis after CORT and EOP intervention. In simple terms, cells were collected at the end of treatment and then incubated with Annexin V and PI dye to avoid light. Flow cytometry was used to detect apoptosis in each group.

### 2.13 ROS detection

At the end of the experiment, ROS levels in cells were detected with dihydroethidium (DHE) probe. DHE can be oxidized by intracellular ROS, and its oxidation products can be incorporated into chromosomal DNA to produce red fluorescence. Therefore, according to the production of red fluorescence in living cells, the amount and change of ROS content in cells can be determined. In simple terms, after cell intervention, 10 μM DHE solution was added and incubated for 30min in dark, then the excess DHE staining solution was washed away. The intensity and change of red fluorescence in cells were detected by laser confocal and flow cytometry.

### 2.14 Western blotting (WB)

After the intervention, the total protein of the cells was extracted. The concentration of total protein was measured and denaturized by boiling water to prepare protein samples for WB experiment. Then the expression of p-Akt, AKT, p-PI3K and PI3K in cells was detected according to the standard steps of WB experiment. In addition, we also extracted the nuclear and cellular proteins of the cells to detect the effect of EOP intervention on NRF2 expression.

### 2.15 Immunofluorescence (IF)

The cells were seeded into laser confocal dishes and treated with EOP and CORT after the cells were completely adhered to the wall. At the end of treatment, cell culture medium was discarded and fixation solution was added to fix the cells, followed by immunofluorescence blocking solution, and the cells were sealed at room temperature for 1 h. At the end of sealing, corresponding primary antibodies (p-Akt, AKT, p-PI3K, PI3K, Nrf2) were added into the small dish and incubated overnight. On the second day, after the primary antibody was discarded, the corresponding fluorescent secondary antibody was added and incubated at room temperature to avoid light for 1 h. Finally, the fluorescence changes in the cells were observed by confocal laser microscopy. The same method was used to detect IF in the brain tissue, and the expression of PI3K, Akt, p-PI3K and p-Akt in the hippocampus of mice was detected by immunofluorescence method.

### 2.16 GC-MS analysis and compound identification

We utilized the gas chromatography-mass spectrometry (GC-MS) analyzer (Agilent 5975C Gas Chromatograph, Agilent Technologies, United States) and HP-5MS capillary column (30 m × 250 μm × 0.25 μm) to analyze the collected essential oils. High-purity helium gas with flow rate of 1.0 ml/min was used as carrier gas. The sampler and detector were set at 280°C. The standard electron impact mass spectrometry source was set at 230°C. The quadrupole temperature was set at 150°C, and the resolution was set at 100:1. The mass spectrometry was performed in full scan monitoring mode with a scanning range of m/z 12-500. The column temperature was set as 80°C (hold for 2 min), and the programmed temperature was increased from 40°C/min to 225°C (hold for 2 min), and from 3°C/min to 260°C (hold for 0 min) kept for 20 min. The components in EOP were obtained by comparing with those recorded in the mass spectrometry recorded by the National Institute of Standards and Technology (NIST) mass spectral library, and the relative percentage contents of each component were determined by area normalization method.

### 2.17 Network analysis

Network analysis was performed with reference to Zhang et al. (Zhang et al., 2021a). In GeneCards (https://www.genecards.org/) and OMIM (https://omim.org/) databases, “depressive disorder” was used as the key word for retrieval, and the retrieval results were combined to eliminate the repeated targets, which was the depression target database. The Mol files of EOP components were uploaded to Swiss Target Prediction (http://www.swisstargetprediction.ch/) database, and the action targets of these compounds were obtained through directional docking. All targets are combined and the repeated targets are eliminated, which is the target library of EOP. The intersection of depression target library and EOP target library was taken, and these intersection targets were considered to be the main targets of EOP in improving depression. At the same time, the interaction of these intersecting genes was analyzed through the STRING online database (https://cn.string-db.org/). The composition-target and disease-target data pairs were imported into Cytoscape software to construct EOP-composition-depressive disorder network map (EOP-DD), and analyzed the network map, and analyzed the main components and targets that contributed to the network map. Kyoto encyclopedia of genes and genomes (KEGG) analysis were performed on the intersection targets with the help of R language to enrich the gene functions and pathways mainly involved in these intersection targets.

### 2.18 Statistics

The raw data were statistically analyzed using Graph Pad Prism 7.0 software, and all data were expressed as Mean ± Standard deviation (SD). Statistical analysis was performed using a two-tailed Student’s t-test, and *p* ＜ 0.05 was considered statistically significant.

## 3 Results

### 3.1 EOP improved depressive-like and anxiety-like behavior in CORT-induced mice

**FIGURE 1:**
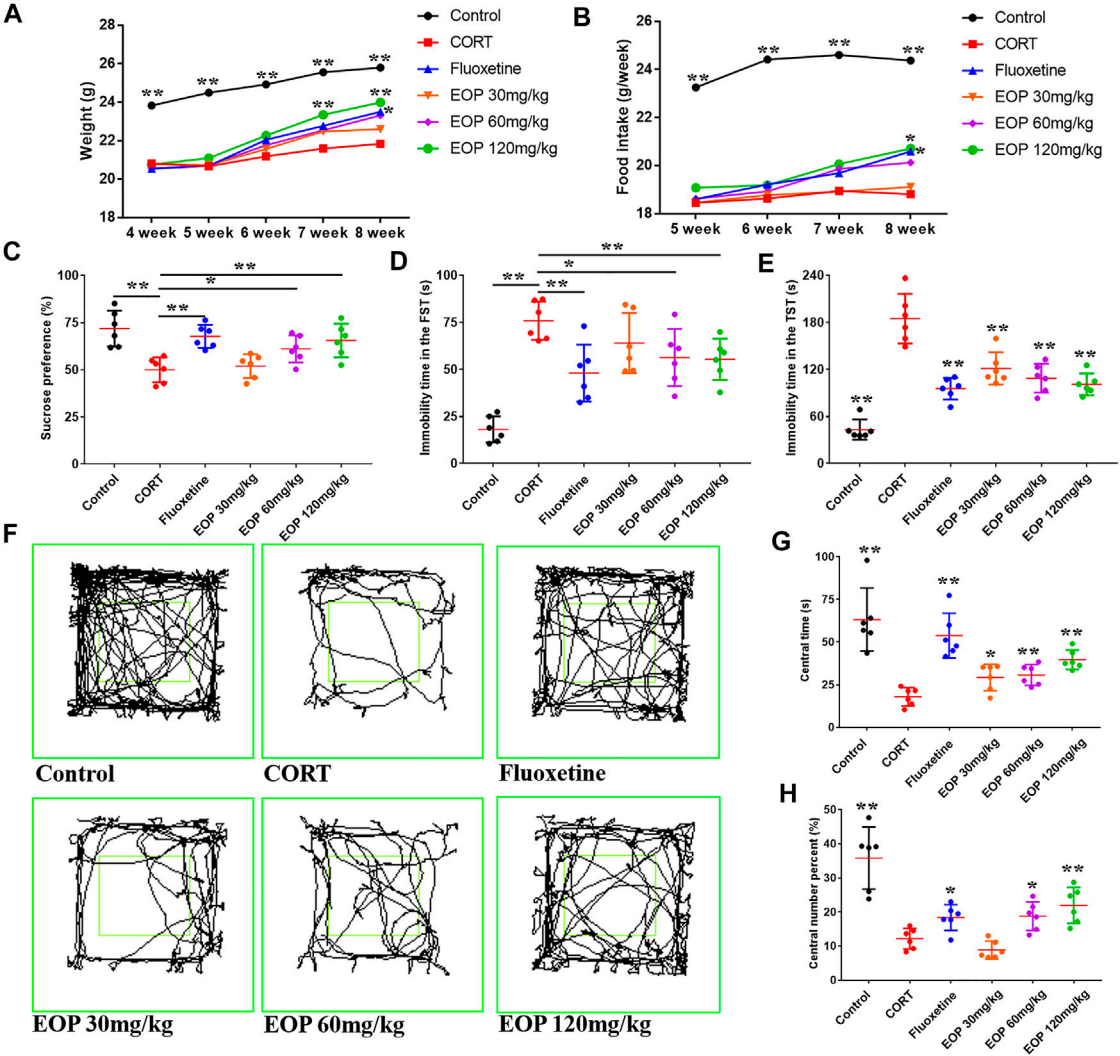
EOP improves basic condition and behavior in corticosterone (CORT)-induced mice **(A)** Changes in body weight, **(B)** changes in food intake, **(C)** changes in sucrose preference percentage (%), **(D)** Forced swimming test (FST) rest time, **(D)** immobility time in tail suspension test (TST), (F) motion track in the open-field test (OFT), (G) time in the central grid of OFT (s), (H) p percent of central grid crossing number in the OFT (%). Data expressed as Mean ± SD, n = 6. **p＜0.01, *p＜0.05 vs*.* CORT group.

After CORT injection, the weight of the mice decreased significantly. As a result, mice in each group weighed less than the control group at the beginning of the drug intervention in the fourth week. EOP and fluoxetine increased body weight in mice after 1 week of treatment (Figure 1A). At week 3, compared with the model group, high-dose EOP significantly increased body weight in mice, while fluoxetine did not increase body weight in mice until week 4 (Figure 1A). We also observed feed intake in mice, which was reduced by CORT injection but increased by fluoxetine and EOP treatment. At week 4, high-dose EOP and fluoxetine significantly increased food intake in mice (Figure 1B). Mice injected with CORT also showed significant depression-like behavior in SPT, FST and TST, decreased SPT, and increased inactivity time in FST and TST experiments. As expected, fluoxetine treatment improved these depressive behaviors in mice. Similarly, fluoxetine showed comparable antidepressant activity at moderate and high doses of EOP. Although 30 mg/kg EOP did not significantly improve the SPT and FST in depressed mice, it also significantly reduced the immobility time in TST (Figures 1C–E). In OFT, the antidepressant effects of EOP and fluoxetine can be evaluated by observing the movement trajectory of mice (Figure 1F). After CORT injection, the percentage and time of crossing in the central grid were significantly reduced in the model group compared with the control group (Figures 1G,H). Fluoxetine can obviously reverse the change of avoidance behavior in depressed mice. EOP also showed a similar improvement, with three different doses of EOP significantly increasing the time spent in the central grid. However, compared with the model group, low dose EOP did not significantly improve the percentage of crossovers in the central grid (Figures 1G,H).

### 3.2 EOP protects CORT-induced hippocampal neuron damage in mice

**FIGURE 2:**
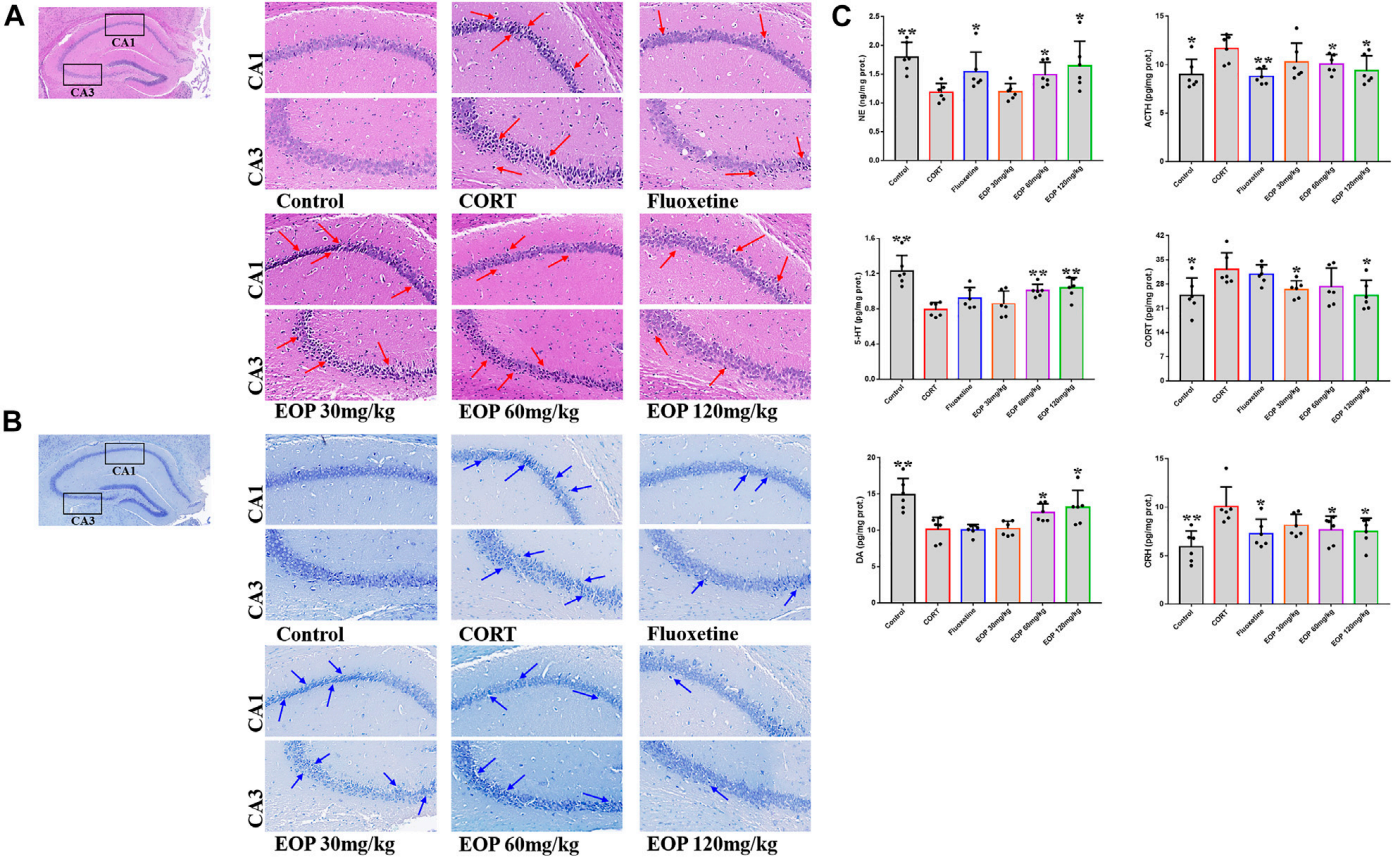
Effects of EOP on the morphology and structure of hippocampal neurons in mice **(A)** Hippocampal HE staining (400X), **(B)** hippocampal Nissl degeneration staining (400X), (C) changes of neurotransmitters in mouse brain tissue. Data expressed as Mean ± SD, n = 3, **p＜0.01, *p＜0.05 vs. CORT group.

As shown in Figure 2A, compared with the control group, hippocampal neurons in CA1 and CA3 areas of mice in CORT model group were sparse, and the number of cell layers was significantly reduced, blurred, and cells atrophied and fragmented, suggesting that CORT could cause significant damage to hippocampal neurons in mice. Fortunately, fluoxetine and EOP therapy reversed these pathological changes, suggesting that EOP and fluoxetine had a good protective effect against hippocampal neuron damage in depressed mice. In addition, Nissl staining of mouse brain tissue also showed that CORT injection could reduce the volume of hippocampal cells in CA1 and CA3 areas of mice, and significantly reduce the cytoplasmic Nissl body (Figure 2B). After fluoxetine and EOP treatment, the morphology and volume of hippocampal nerve cells were restored to a certain extent, and the number of cytoplasmic Nissl increased. In conclusion, EOP protects against CORT-induced hippocampal neuron damage in mice.

### 3.3 EOP regulates transmitter levels in mouse brain tissue

As shown in Figure 2C, after CORT injection, the content of ACTH in mouse brain tissue was significantly higher than that in the control group, while the content of NE was significantly lower. Fluoxetine and EOP treatment both reduced ACTH levels and increased NE levels, but low doses of EOP did not seem to affect the levels of these neurotransmitters. After CORT injection, the contents of ACH and CORT in the brain tissues of mice increased significantly compared with the control group, while the levels of 5-HT and DA decreased significantly. EOP treatment alleviated the changes. Fluoxetine could reduce the level of ACH, but had no significant effect on the levels of 5-HT, CORT and DA. In addition, we also examined changes in CRH levels in the mouse brain tissue. Compared with the control group, the level of CRH in CORT group was significantly increased, and both fluoxetine and EOP could significantly reduce the level of CRH in CORT group. These results indicate that EOP treatment can effectively reduce the over release of ACTH, ACH and CORT, and increase the level of NE, 5-HT and DA neurotransmitter in the brain tissues of depressed mice.

### 3.4 EOP protects CORT-induced PC12 cell damage

**FIGURE 3:**
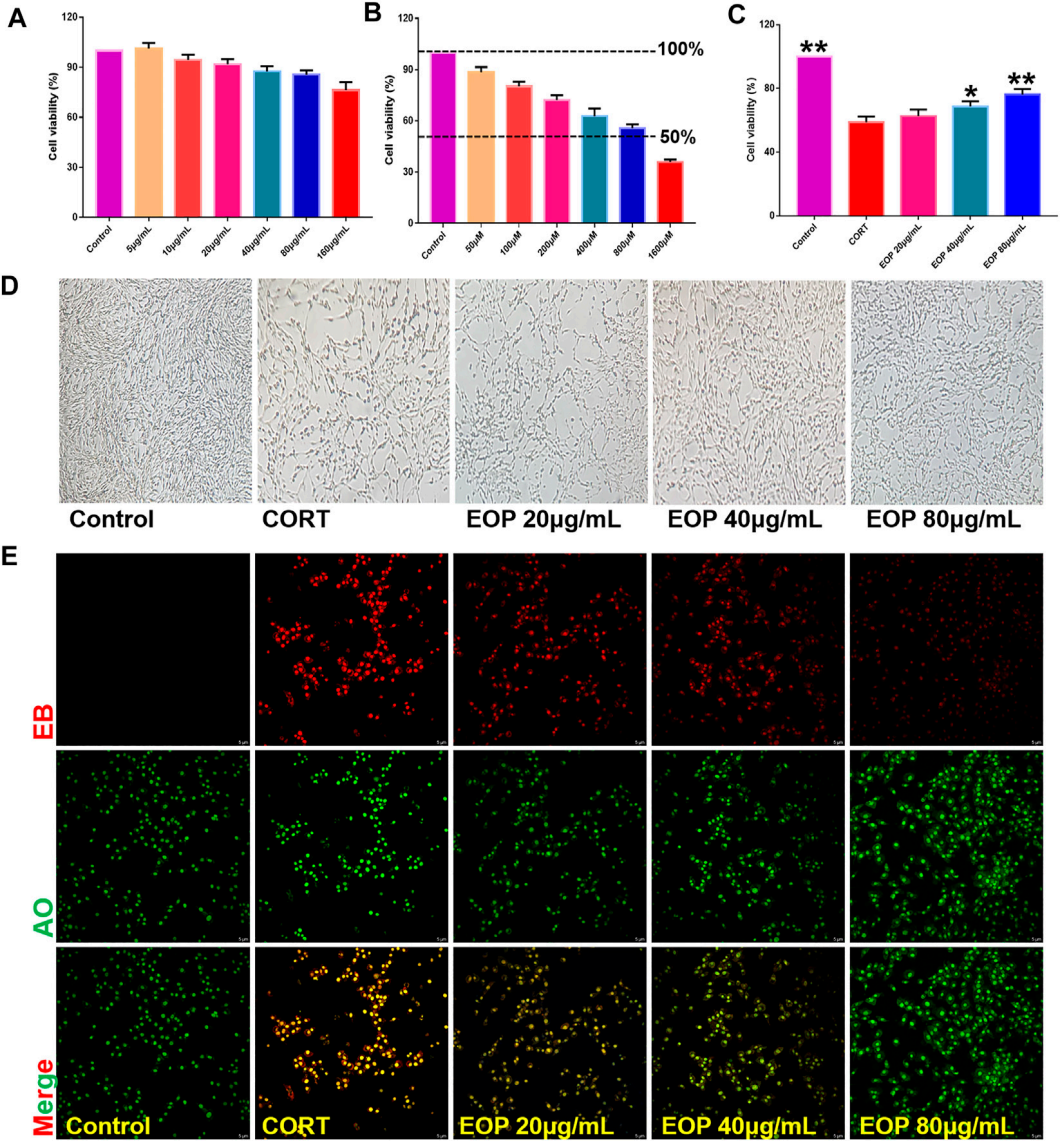
EOP reduced the effect of CORT on the viability of PC12 cells **(A)** Effects of different concentrations of EOP on the viability of PC12 cells. **(B)** Effects of different concentrations of CORT on the viability of PC12 cells (C) Effects of EOP on the viability of PC12 cells treated with CORT. (D) Morphological changes of PC12 cells treated with EOP and CORT. (E) AO-EB staining representative diagram of PC12 cells after EOP and CORT intervention. Data expressed as Mean ± SD, n = 3, **p＜0.01, *p＜0.05 vs. CORT group.

**FIGURE 4:**
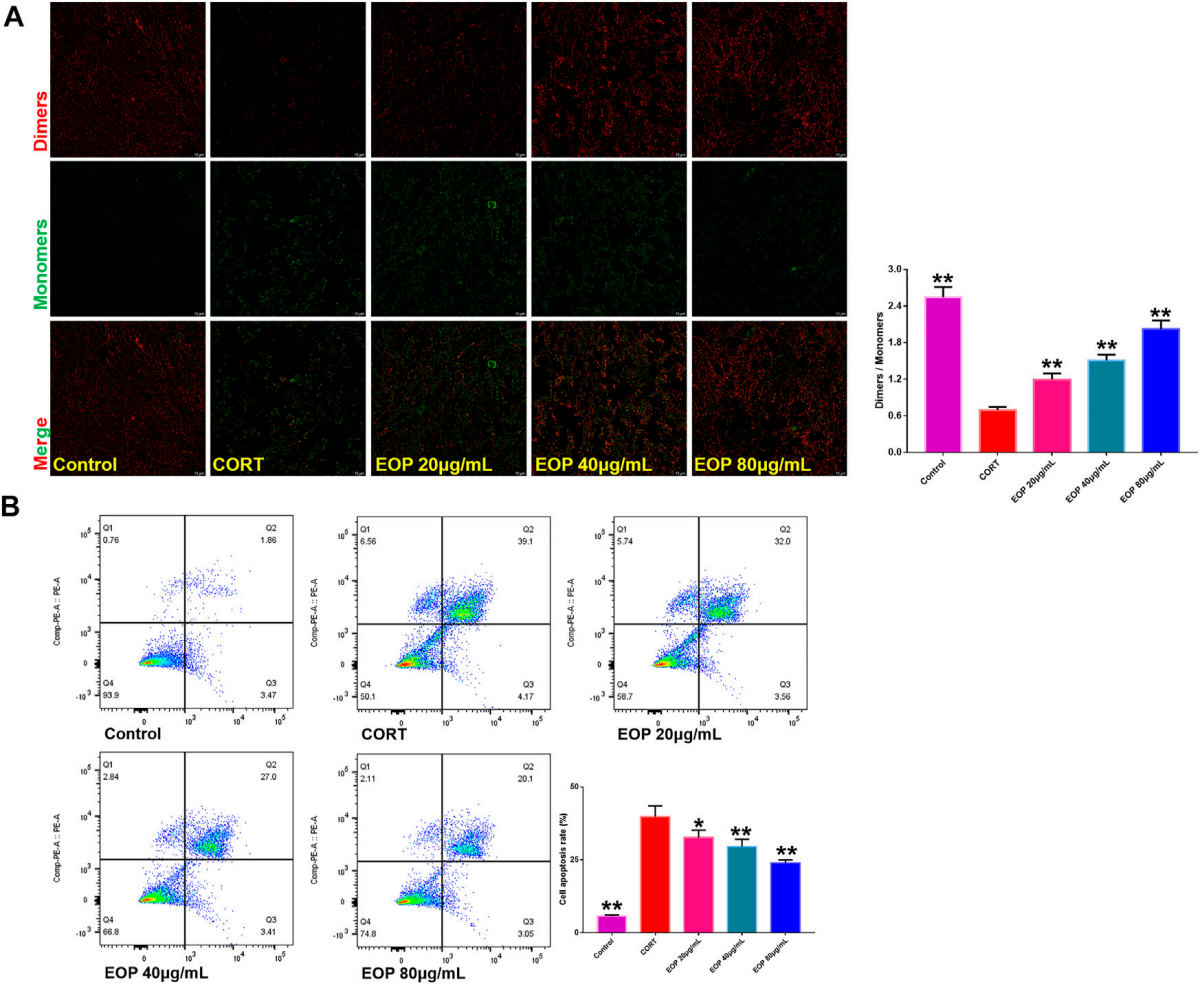
EOP inhibits CORT-induced apoptosis of PC12 cells **(A)** Effects of EOP on CORT-induced cell membrane potential collapse of PC12 cells. **(B)** Effects of EOP on CORT-induced apoptosis of PC12 cells. Data expressed as Mean ± SD, n = 3. **p＜0.01, *p＜0.05 vs. CORT group.

In order to further evaluate the effect of EOP on nerve injury caused by depression, an *in vitro* CORT-induced PC12 cell injury model was established to evaluate the neuroprotective effect of EOP. The optimal concentrations of EOP and CORT were screened by CCK8 experiment. Results as shown in Figure 3A, when the concentration of EOP was less than 80 μg/ml, the survival rate of cells was above 80% after 24 h of intervention, suggesting that there was no cytotoxicity to PC12 when the concentration of EOP was less than 80 μg/ml. Meanwhile, we also evaluated the effect of CORT on PC12 cell viability. Results as shown in Figure 3B, when CORT concentration exceeded 800 μM, cell viability decreased to about 50% after 24 h of intervention. In summary, we selected EOP of 20 μg/ml, 40 μg/ml and 80 μg/ml to pre-intervene PC12 cells for 2 h, and then added 800 μM CORT for 24 h to evaluate the protective effect of EOP on PC12 cells. Similarly, we also tested the survival rate of PC12 cells in the presence of EOP and CORT. Results as shown in Figures 3C,D, CORT intervention significantly reduced the survival rate of PC12 cells, while EOP treatment significantly reversed this situation. At the same time, we also used AO-EB staining to evaluate the protective effect of EOP on PC12 cells. As shown in Figure 3E, after CORT intervention, EB staining positive cells were relatively increased, and red fluorescence staining was deepened, suggesting that CORT would cause damage to the cell membrane of PC12 cells and increase apoptosis. EOP treatment can improve this phenomenon and reduce the intensity of EB fluorescence staining and the number of positive staining cells. Similarly, the detection of MMOP of PC12 cells also obtained similar results. CORT intervention significantly increased the number of JC-1 monomers and decreased the number of JC-1 polymers, but pre-treatment with EOP inhibited the formation of JC-1 monomers and alleviated the breakdown of MMOP caused by CORT (Figure 4A). Finally, to further determine whether the protective effect of EOP on CORT-induced PC12 cell damage was related to apoptosis, flow cytometry was used to measure the apoptosis rate of each group at the end of intervention. As expected, CORT induced PC12 cell apoptosis significantly, while EOP treatment significantly inhibited PC12 cell apoptosis (Figure 4B). In conclusion, our results suggest that EOP also has a significant protective effect on CORT-induced PC12 cell damage *in vitro*, and this protective effect is related to inhibition of cell apoptosis.

### 3.5 EOP improves CORT-induced oxidative stress in PC12 cells

**FIGURE 5:**
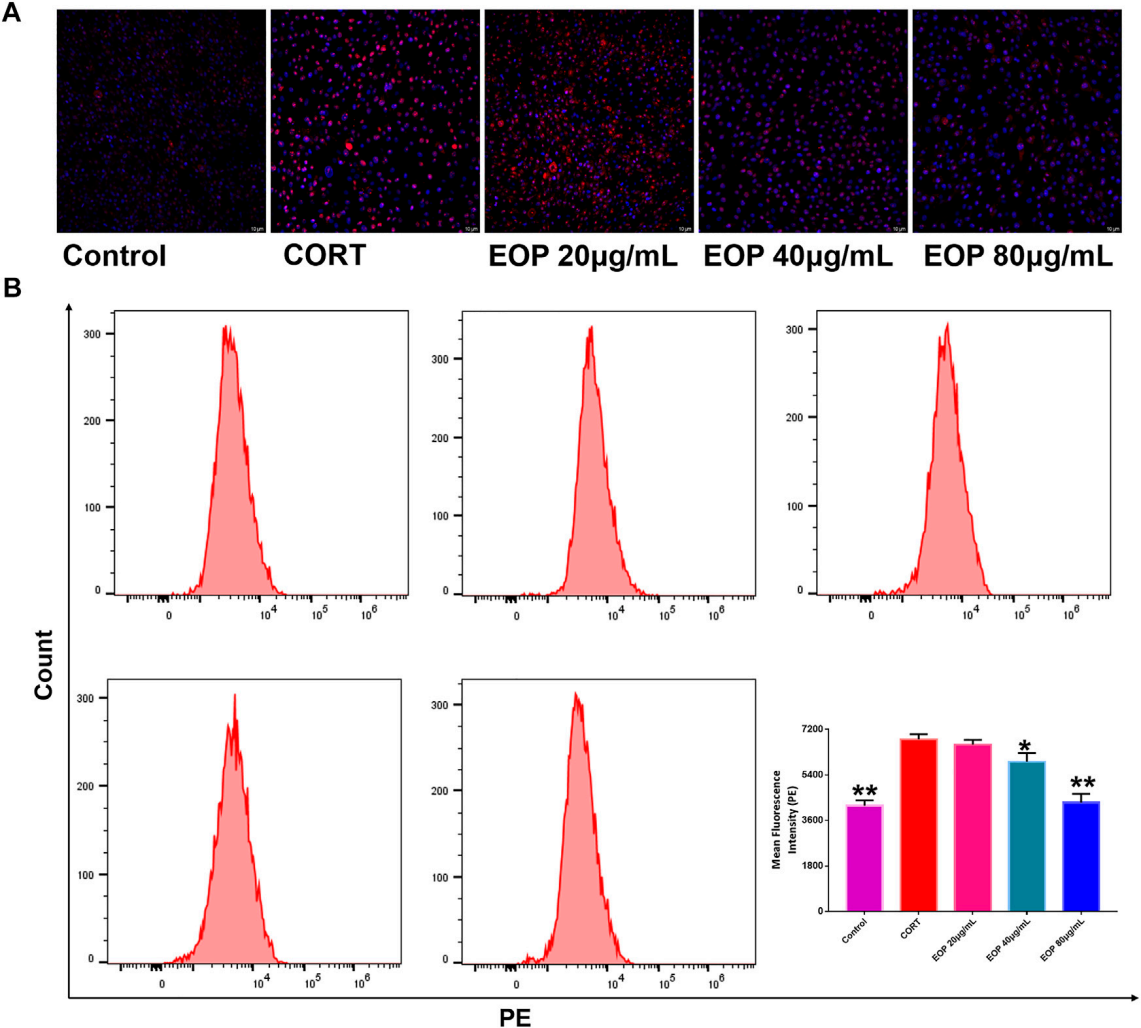
EOP inhibited CORT-induced oxidative stress in PC12 cells **(A)** Laser confocal observation of EOP improved ROS production in PC12 cells, **(B)** flow cytometry analysis of ROS changes in PC12 cells. Data expressed as Mean ± SD, n = 3. **p＜0.01, *p＜0.05 vs. CORT group.

Many studies have found that oxidative stress is one of the main causes of neurological damage in depression. Therefore, we also evaluated the effect of EOP on oxidative stress state in PC12 cells. Results as shown in Figure 5A, compared with the control group, the number of ROS positive staining cells in CORT group was significantly increased, indicating that CORT can significantly increase ROS production in PC12 cells. However, the number of ROS positive color cells in each dose of EOP group decreased significantly, suggesting that EOP can inhibit the production of ROS in CORT-induced PC12 cells. Similar results were obtained by measuring ROS levels in cells by flow cytometry (Similar results were obtained by measuring ROS levels in cells by flow cytometry (Figure 5B).

### 3.6Analysis of the constituents of volatile oil obtained from EOP

The composition of EOP was analyzed with the help of GC-MS (Supplementary Figure S1), and finally 21 components were identified (Qu et al., 2009; Fan et al., 2012; Zhao et al., 2015). Including tetradecane, pentadecane, myrtanal, hexadecanoic acid, methyl ester, 2-heptadecanone, hexadecanoic acid, ethyl ester, paeonol, lauric acid, methyl linoleate, tetradecanoic acid, dibutyl phthalate, myristelaidic acid, pentadecanoic acid, n-hexadecanoic acid, palmitoleic acid, heptadecanoic acid, cis-10-heptadecenoic acid, stearic acid, oleic acid, linoleic acid, linolenic acid is shown in Table 1.

**TABLE 1:** Compounds identified in volatile oil from EOP.

Peak no.	RT (min)	Compound	Formula	Molecular weight	Relative amount (%)
1	4.058	Tetradecane	C_14_H_30_	198.39	0.02
2	4.517	Pentadecane	C_15_H_32_	212.41	0.06
3	4.869	Myrtanal	C_10_H_16_O	152.23	0.12
4	7.078	Hexadecanoic acid, methyl ester	C_17_H_34_O_2_	270.5	0.27
5	7.174	2-Heptadecanone	C_17_H_34_O	254.5	0.18
6	7.238	Hexadecanoic acid, ethyl ester	C_18_H_36_O_2_	284.5	0.10
7	7.451	Paeonol	C_9_H_10_O_3_	166.17	0.69
8	8.635	Lauric acid	C_12_H_24_O_2_	200.32	0.31
9	8.763	Methyl linoleate	C_19_H_34_O_2_	294.5	0.21
10	10.524	Tetradecanoic acid	C_14_H_28_O_2_	228.37	1.24
11	10.705	Dibutyl phthalate	C_16_H_22_O_4_	278.34	0.54
12	11.068	Myristelaidic acid	C_14_H_26_O_2_	226.35	0.20
13	11.719	Pentadecanoic acid	C_15_H_30_O_2_	242.4	1.51
14	13.148	n-Hexadecanoic acid	C_16_H_32_O_2_	256.42	37.80
15	13.660	Palmitoleic acid	C_16_H_30_O_2_	254.41	4.89
16	14.620	Heptadecanoic acid	C_17_H_34_O_2_	270.5	0.70
17	15.250	cis-10-Heptadecenoic acid	C_17_H_32_O_2_	268.4	1.00
18	16.242	Stearic acid	C_18_H_36_O_2_	284.5	0.68
19	16.754	Oleic acid	C_18_H_34_O_2_	282.5	6.3
20	17.693	Linoleic acid	C_18_H_32_O_2_	280.4	29.50
21	18.856	Linolenic acid	C_18_H_30_O_2_	278.4	4.58

### 3.7 The underlying mechanism by which EOP ameliorates depression is related to the PI3K-Akt pathway

**FIGURE 6:**
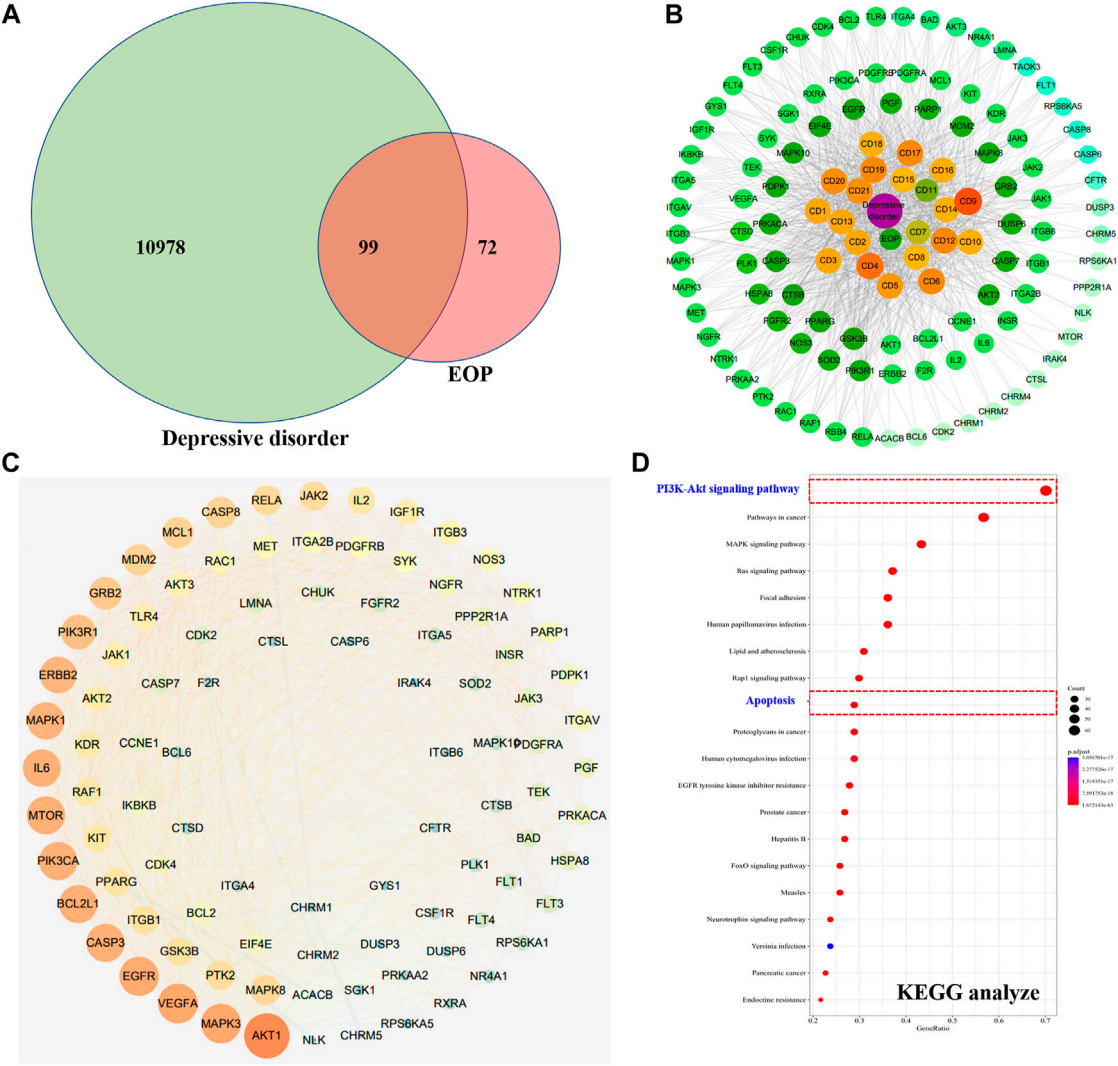
Prediction of antidepressant effects of EOP by network pharmacology **(A)** Intersection of the depression target library and the EOP target library. **(B)** EOP-composition-depressive disorder network map (EOP-DD) (C) Interaction network diagram of intersection gene (PPI). (D) KEGG enrichment results of intersection genes.

A total of 11,077 targets related to depression were obtained through GeneCards and OMIM database retrieval. A total of 171 targets acted by EOP components were predicted by reverse docking of Swiss Target Prediction database. The intersection of EOP targets and depression-related targets was processed, and 99 intersection targets were obtained, which were considered as the main targets of EOP to improve depression (Figures 6A,C). In addition, we also constructed the composition-target network diagram of EOP to improve depression. Through the network diagram analysis, we found that 21 components in EOP were involved in its antidepressant effect, among which CD9, CD4, CD6, CD12, CD17 and other components may be the main active components of EOP to exert antidepressant effect (Figure 6B, Supplementary Table S1). Finally, the main mechanism of EOP improving depression was predicted by KEGG analysis of the intersection targets. The results showed that the targets of EOP were mainly enriched in the PI3K-Akt pathway and apoptosis, suggesting that the potential mechanism of EOP improving depression is related to the PI3K-Akt pathway (Figure 6D).

### 3.8 The antidepressant effect of EOP *in Vitro* and *in Vivo* is related to the activation of PI3K-Akt

**FIGURE 7:**
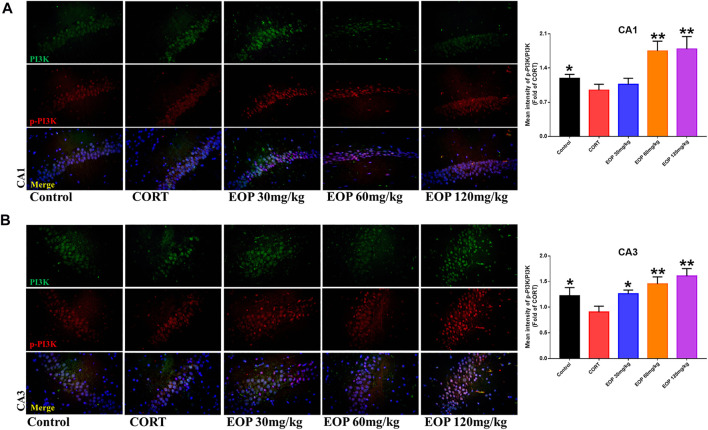
EOP treatment induced PI3K phosphorylation in hippocampal neurons of CORT-induced depressed mice, n = 3 **(A)** The changes of PI3K phosphorylation in hippocampal CA1 neurons were observed by immunofluorescence (IF). **(B)** The changes of PI3K phosphorylation in hippocampal CA3 neurons were observed by IF. **p＜0.01, *p＜0.05 vs. CORT group.

**FIGURE 8:**
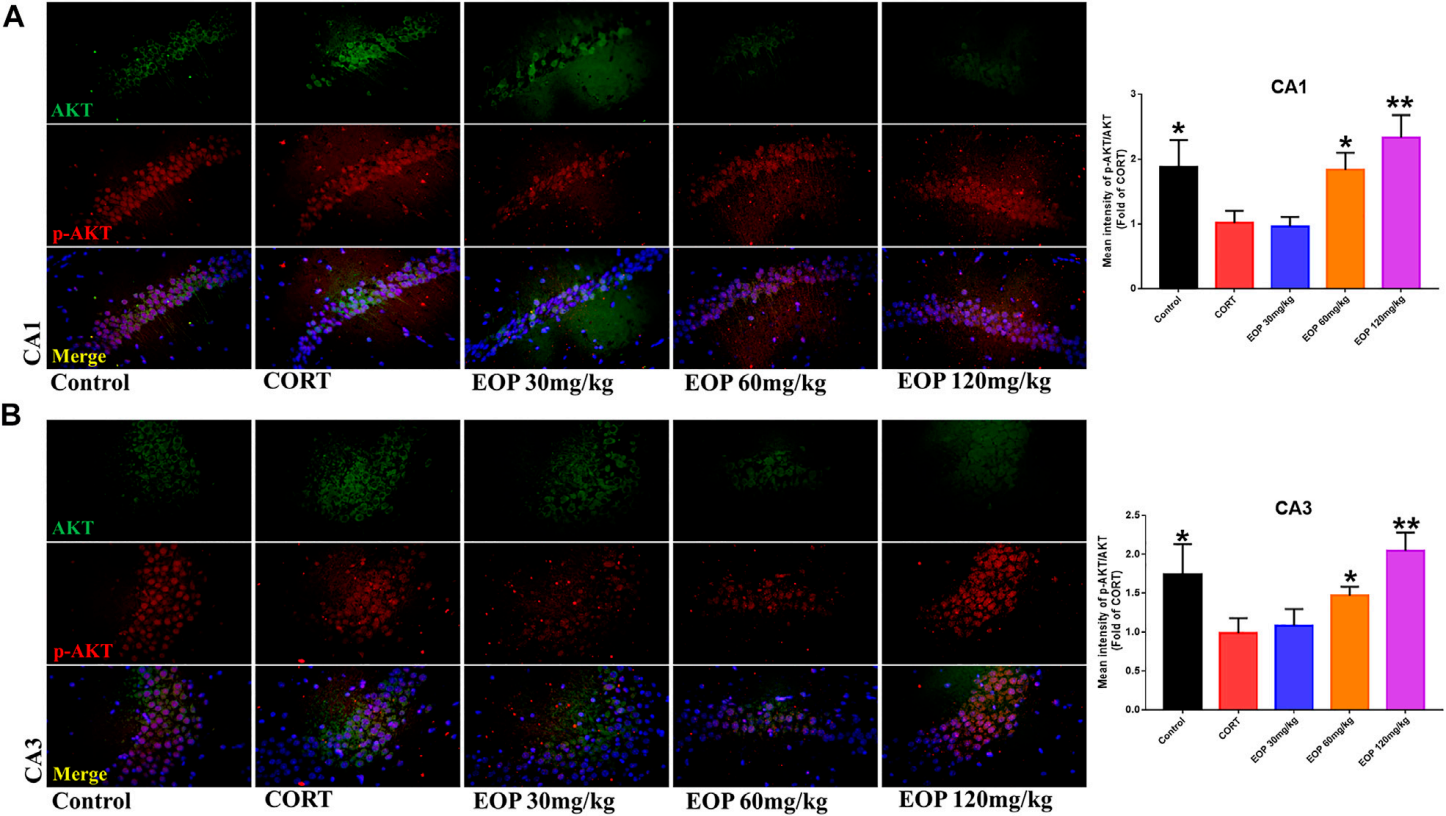
EOP treatment induced AKT phosphorylation in hippocampal neurons of CORT-induced depressed mice, n = 3 **(A)** The changes of AKT phosphorylation in hippocampal CA1 neurons were observed by immunofluorescence (IF). **(B)** The changes of AKT phosphorylation in hippocampal CA3 neurons were observed by IF. **p＜0.01, *p＜0.05 vs. CORT group.

**FIGURE 9:**
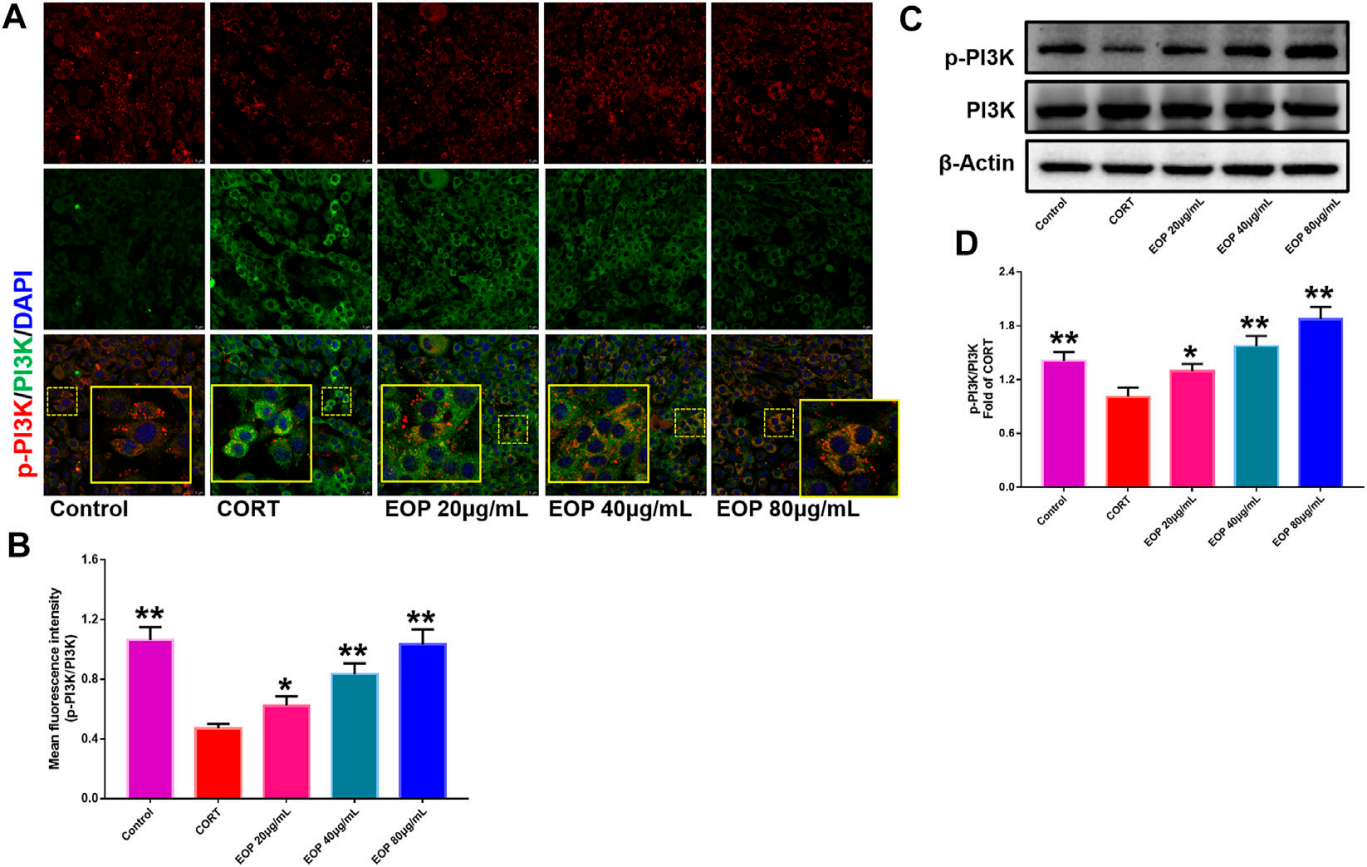
EOP promoted the phosphorylation of PI3K in CORT-induced PC12 cells **(A)** Immunofluorescence (IF) assay demonstrated that EOP promoted the phosphorylation of PI3K in CORT-induced PC12 cells **(B)** The statistical results of the fluorescence intensity of the IF experiment. **(C)** WB was used to detect the effect of EOP on the phosphorylation of PI3K in CORT-induced PC12 cells. **(D)** Statistical results of the WB experiment. Data expressed as Mean ± SD, n = 3. **p＜0.01, *p＜0.05 vs. CORT group.

**FIGURE 10:**
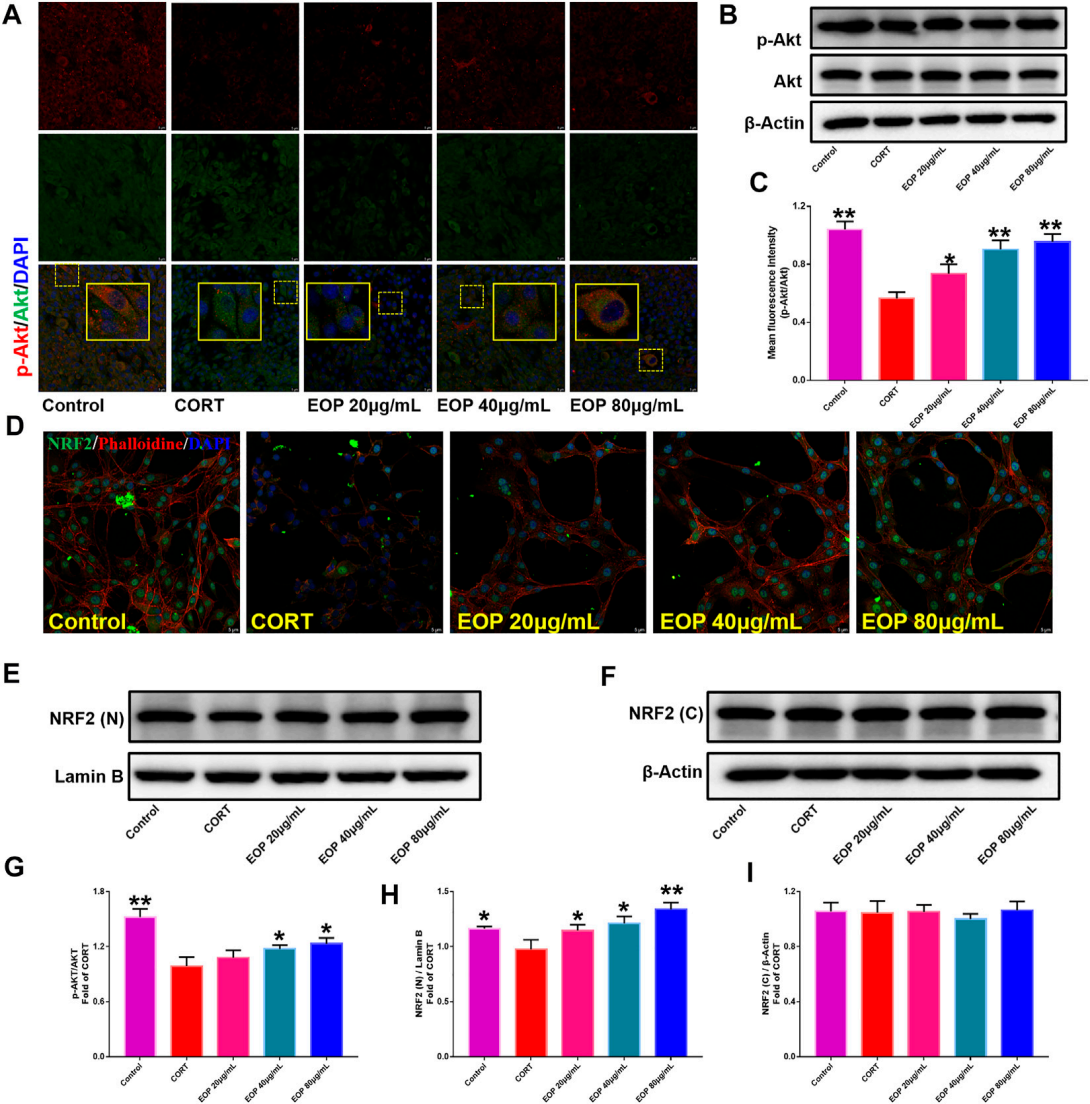
EOP promoted the phosphorylation of AKT and nuclear transcription of Nrf2 in CORT-induced PC12 cells. **(A)** Immunofluorescence (IF) assay demonstrated that EOP promoted the phosphorylation of AKT in CORT-induced PC12 cells. **(B,G)** WB was used to detect the effect of EOP on the phosphorylation of AKT in CORT-induced PC12 cells. **(C)** Statistical results of the IF experiment **(D)** IF assay confirmed that EOP promoted the nuclear transcription of Nrf2 in CORT-induced PC12 cells. **(E–I)** WB was used to detect the effect of EOP on CORT-induced NRF2 expression in the nucleus and cytoplasm of PC12 cells. Data expressed as Mean ± SD, n = 3. * *p＜0.01, *p＜0.05 vs. CORT group.

EOP has been confirmed to have significant anti-depressive activity *in vivo* and can protect hippocampal neuron cells from injury. *In vitro* experiments, we obtained similar results. EOP treatment can inhibit CORT-induced PC12 cell damage, inhibit MMOP collapse, reduce ROS level in PC12 cells, and inhibit cell apoptosis. It was predicted in network analysis that the mechanism of EOP improving depression might be related to apoptosis and the PI3K-Akt pathway, which was consistent with previous *in vitro* and *in vivo* results. Meanwhile, the changes of PI3K-Akt pathway in the hippocampal tissues of mice after EOP intervention were detected by IF staining. Results as shown in Figure 7, compared with the control group, CORT intervention inhibited the relative expression of p-PI3K in hippocampal neurons in CA1, CA3 region and increased the expression of PI3K. Interestingly, after EOP treatment, the fluorescence expression of p-PI3K in hippocampal neurons was significantly enhanced, while that of PI3K was weakened. Similarly, Figure 8 shows the expression of AKT in hippocampal neurons. EOP treatment can reverse the CORT-induced decrease in p-Akt expression and increase in AKT expression. We also detected the activation of PI3K/AKT pathway in CORT-induced PC12 cells. Results as shown in Figures 9, 10, CORT intervention significantly inhibited the phosphorylation of PI3K and AKT. Fortunately, EOP therapy activates the PI3K/AKT pathway and increases the phosphorylation of PI3K and AKT. In addition, a large number of studies have reported that Nrf2 is an important downstream target in the PI3K/AKT pathway, and plays an important role in regulating the level of intracellular oxidative stress (Zhang et al., 2021b). Therefore, we also detected Nrf2 expression changes in PC12 cells. As shown in Figures 10D–I, CORT intervention inhibited the nuclear transcription of Nrf2 in PC12 cells compared with the control group. The inhibition of Nrf2 nuclear transcription induced by CORT was alleviated by EOP therapy. In conclusion, through *in vitro* and *in vivo* activity studies combined with network analysis, it is clear that EOP has significant anti-depressive activity, which can protect neuronal cell damage caused by depression, inhibit neuronal cell apoptosis, and reduce the level of intracellular oxidative stress. Meanwhile, these pharmacological activities of EOP are closely related to the activation of PI3K/AKT/NRF2 pathway in neuronal cells.

## 5 Discussions

In Chinese medicinal theory, mental or emotional disorders including depression, anxiety and affective disorders are mainly attributed to the stagnation of liver-*Qi* (Expert Group of Psychosomatic Medicine Branch of China Association of Chinese Medicine, 2021; Zhang et al., 2022). Consequently, many of the herbal medicines have the functions of “*Relieving Qi stagnancy in liver*” could be used to treat depression, such as the roots of *P. lactiflora*, whole herbs of *Bupleurum chinense*, and the herbs of *Acorus tatarinowii*, *etc* (Xu et al., 2011; Han et al., 2013; Sun et al., 2021; Zhu et al., 2022). In clinical practice, the roots of *P. lactiflora* is commonly used in traditional Chinese medicine to treat depression. And modern pharmacological studies have found that the roots of P. lactiflora and its monomeric components have obvious antidepressant effects *in vitro* and *in vivo*. Their antidepressant effects are thought to be related to the regulation of neurotransmitters, neurotrophic factors, glucocorticoid receptor activity, and hypothalamic-pituitary-adrenal function (Wang et al., 2011; Jin et al., 2016; Li Y. C. et al., 2020). There is a large amount of volatile oil in *Radix Paeoniae Alba.* (EOP), and the EOP can be extracted by steam distillation, and the yield can reach 1.0%. So far, more than fifty EOP have been reported. At the same time, there are also many reports that the EOP is one of the main active components of *Radix Paeoniae Alba.* In our present study, we have firstly systemically studied the anti-depression effects of EOP, and explored the potential mechanisms corresponding to its anti-depression effects.

Cortisol, also known as the stress hormone, plays an important role in the development of affective disorders especially in depression (Geiger et al., 2015; Keller et al., 2017). In addition, the hypothalamic–pituitary–adrenal (HPA) axis plays an important role in the regulation of cortisol, and it is also found that changes in HPA axis with increased amount of plasma cortisol is one of the biological features in severe depression patients (Keller et al., 2017; Malhi and Mann, 2018). For investigation of the depression, corticosterone (CORT) is commonly used to induce chronic depression animals with disorders in HPA axis function with high levels of cortisol and glucocorticoids in blood plasm which are similar to the patients with depression (Malhi and Mann, 2018; Zhou et al., 2021; Oliveira et al., 2022). In our present study, we have successfully prepared the depression mice with CORT injection (i.p.), and we found that EOP has obvious anti-depression effects against the depression symptoms of CORT induced mice (decreasing the immobility time in FST and TST, and increasing sucrose consumption and locomotor activity). HPA axis function disorder is closely related to the high level of cortisol of depression patients, and there are three important hormones in HPA axis including corticotropin-releasing hormone (CRH), corticotropin (ACTH) and cortisol. CRH and ACTH are commonly increased for the depression patients, and play important roles for the imbalance of cortisol (Malhi and Mann, 2018; Dwyer et al., 2020). In our present study, we found that EOP can significantly down-regulate the levels of CRH, ACTH, and cortisol in mouse brain tissue. Previous studies have suggested that low levels of monoamine neurotransmitters, including 5-hydroxytryptamine (5-HT), noradrenaline (NE), and dopamine (DA), are commonly found in the plasm of depression patients. Importantly, these monoamine neurotransmitters are also used as the targets for many of the current available clinical antidepressants for treating depression (Boku et al., 2018; Malhi and Mann, 2018). In our present work, we found that EOP treatment could increase the levels of monoamine neurotransmitters including 5-HT, NE, and DA in CORT induced depression mice.

It is reported that depression would damage the hippocampal neurons both in experimental depression animals and clinical depression patients (Malhi and Mann, 2018; Xu et al., 2021). Our present results showed that EOP treatment could alleviate the CORT induced hippocampal neurons damage in mice. However, it is not clear how the EOP protects hippocampal neurons from damage in CORT induced mice. Consequently, we further explored the possible protective way and molecular mechanisms of EOP for its protective effects on neuron cells using the CORT induced PC12 cell model. Our results revealed that EOP can increase the cell proliferation and cell viability of PC12 cells by suppression of apoptosis induced by CORT. Furthermore, wo explored the further molecular mechanisms corresponding for its inhibitory effect against apoptosis induced by CORT. By using the network analysis method, it is revealed that the phosphoinositide-3-kinase (PI3K)-protein kinase B (Akt) might be one of the predominant singnalling pathway for the anti-apoptotic effects of EOP on hippocampal neurons induced by CORT. The PI3K/Akt is an important signaling pathway for the proliferation, survival, and differentiation of various types of cells, and also plays a crucial effect in the regulation of cell apoptosis (Zhou et al., 2009; Sun et al., 2012; Li R. L. et al., 2020; Zhang et al., 2021b). In normal condition, upstream signaling firstly activates the PI3K proteins via promoting its phosphorylation, then the activated PI3K can promote the phosphorylation and activation of its down-stream signaling molecule of Akt. The activated Akt can subsequently induce the ubiquitination and hydrolysis of the pro-apoptotic proteins such as Bax and Bad, resulting in the suppression of cell apoptosis. In addition, the activated Akt could also promote the nuclear transcription of Nrf2. Then the intranuclear Nrf2 can combined to the antioxidant response element (ARE) and subsequently result in the productions of a series of antioxidant enzymes, such as NQO1, HO-1, SOD, GSH, etc. These antioxidant enzymes could further scavenge the reactive oxygen species (ROS) to protect the cell from apoptosis induced by ROS (Song et al., 2017; Xiao et al., 2018; Yuan et al., 2020; Zhang et al., 2021c). In our present study, we found that EOP can upregulate the phosphorylation of PI3K and Akt both in hippocampal neurons of mice induced by CORT and PC12 cells induced by CORT, and can also promote the nuclear transcription of Nrf2 in PC12 cells induced by CORT. All these results mentioned above suggested that EOP might exert its anti-apoptosis effects on hippocampal neurons via PI3K/Akt/Nrf2 signaling pathway.

In conclusion, by using integrated approach based on network analysis combined with experimental verification, our present study revealed that the essential oil from the roots of *Paeonia lactiflora* has protective effect against corticosterone-induced depression in mice via modulation of PI3K/Akt signaling pathway. Our present study would be beneficial for the further development of EOP as a clinical anti-depressant.

## Data Availability

The datasets presented in this study can be found in online repositories. The names of the repository/repositories and accession number(s) can be found in the article/Supplementary Material.
