# Supplementary material for: Essential oil from the roots of Paeonia lactiflora pall. has protective effect against corticosterone-induced depression in mice via modulation of PI3K/Akt signaling pathway
Source: Front Pharmacol. 2022 Sep 16;13:999712. doi: 10.3389/fphar.2022.999712 (PMC9523509; doi:10.3389/fphar.2022.999712)
Supplement: Supplementary file 1 [file Table1.docx]

Table S1 Topological parameter analysis of the composition-target network diagram of EOP to improve depression.

| Name | Degree | Average Shortest Path Length | Betweenness Centrality | Closeness Centrality |
| --- | --- | --- | --- | --- |
| CD9 | 54 | 1.958333 | 0.04217 | 0.510638 |
| CD4 | 50 | 1.991667 | 0.037178 | 0.502092 |
| CD6 | 47 | 2.058333 | 0.027318 | 0.48583 |
| CD12 | 47 | 2.041667 | 0.02984 | 0.489796 |
| CD17 | 47 | 2.075 | 0.024389 | 0.481928 |
| CD19 | 47 | 2.075 | 0.024837 | 0.481928 |
| CD20 | 46 | 2.075 | 0.025068 | 0.481928 |
| CD21 | 46 | 2.091667 | 0.024428 | 0.478088 |
| CD5 | 45 | 2.091667 | 0.023273 | 0.478088 |
| CD1 | 43 | 2.125 | 0.02157 | 0.470588 |
| CD2 | 43 | 2.125 | 0.021389 | 0.470588 |
| CD3 | 43 | 2.125 | 0.044068 | 0.470588 |
| CD13 | 43 | 2.108333 | 0.023454 | 0.474308 |
| CD10 | 42 | 2.125 | 0.019731 | 0.470588 |
| CD14 | 42 | 2.125 | 0.018456 | 0.470588 |
| CD16 | 42 | 2.141667 | 0.017231 | 0.466926 |
| CD18 | 42 | 2.141667 | 0.017409 | 0.466926 |
| CD8 | 41 | 2.141667 | 0.018072 | 0.466926 |
| CD15 | 41 | 2.158333 | 0.015588 | 0.46332 |
| CD7 | 34 | 2.291667 | 0.025999 | 0.436364 |
