# Supplementary figures and images for: Essential oil from the roots of Paeonia lactiflora pall. has protective effect against corticosterone-induced depression in mice via modulation of PI3K/Akt signaling pathway
Source: Front Pharmacol. 2022 Sep 16;13:999712. doi: 10.3389/fphar.2022.999712 (PMC9523509; doi:10.3389/fphar.2022.999712)

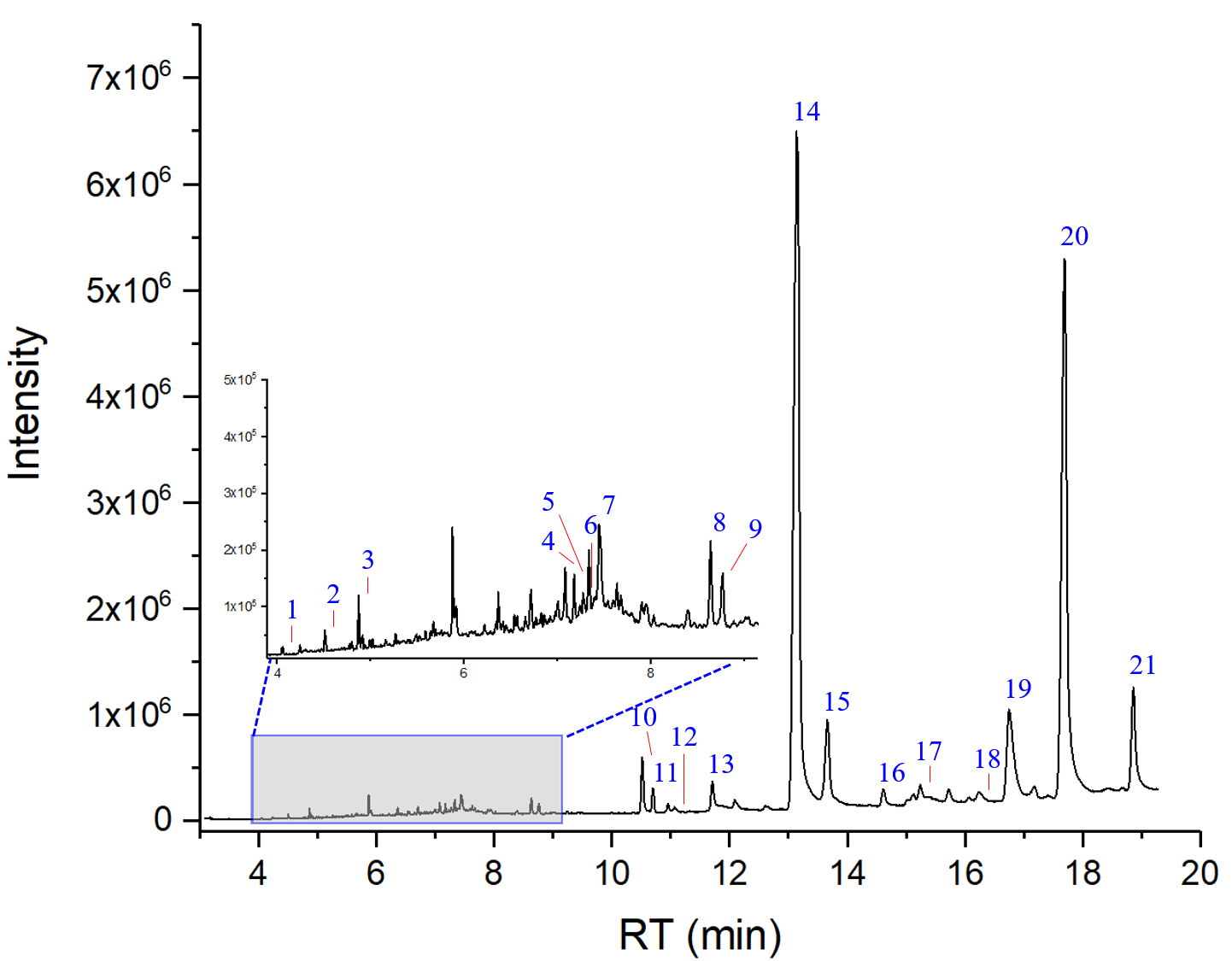

Supplement: Supplementary file 2 [file Image1.TIF]
